# The gut microbiome as a potential source of non-invasive biomarkers of chronic obstructive pulmonary disease

**DOI:** 10.3389/fmicb.2023.1173614

**Published:** 2023-07-25

**Authors:** Naijian Li, Xinzhu Yi, Chiyong Chen, Zhouli Dai, Zhishan Deng, Jinding Pu, Yumin Zhou, Bing Li, Zhang Wang, Pixin Ran

**Affiliations:** ^1^Department of Allergy and Clinical Immunology, State Key Laboratory of Respiratory Disease, National Clinical Research Center for Respiratory Disease, Guangzhou Institute of Respiratory Health, The First Affiliated Hospital of Guangzhou Medical University, Guangzhou, Guangdong, China; ^2^Institute of Ecological Science, School of Life Science, South China Normal University, Guangzhou, Guangdong, China; ^3^The GMU-GIBH Joint School of Life Sciences, Guangzhou Medical University, Guangzhou, Guangdong, China; ^4^College of Medicine, Lishui University, Lishui, Zhejiang, China; ^5^Department of Respiratory Medicine, The Third Affiliated Hospital of Guangzhou Medical University, Guangzhou, Guangdong, China

**Keywords:** COPD, gut microbiome, metagenomics, biomarker, diagnosis

## Abstract

**Background:**

The link between gut microbial dysbiosis and the development of chronic obstructive pulmonary disease (COPD) is of considerable interest. However, little is known regarding the potential for the use of the fecal metagenome for the diagnosis of COPD.

**Methods:**

A total of 80 healthy controls, 31 patients with COPD severity stages I or II, and 49 patients with COPD severity stages III or IV fecal samples were subjected to metagenomic analysis. We characterized the gut microbiome, identified microbial taxonomic and functional markers, and constructed a COPD disease classifier using samples.

**Results:**

The fecal microbial diversity of patients with COPD stages I or II was higher than that of healthy controls, but lower in patients with COPD stages III or IV. Twenty-one, twenty-four, and eleven microbial species, including potential pathogens and pro-inflammatory bacteria, were significantly enriched or depleted in healthy controls, patients with COPD stages I or II, and patients with COPD stages III & IV. The KEGG orthology (KO) gene profiles derived demonstrated notable differences in gut microbial function among the three groups. Moreover, gut microbial taxonomic and functional markers could be used to differentiate patients with COPD from healthy controls, on the basis of areas under receiver operating characteristic curves (AUCs) of 0.8814 and 0.8479, respectively. Notably, the gut microbial taxonomic features differed between healthy individuals and patients in stages I-II COPD, which suggests the utility of fecal metagenomic biomarkers for the diagnosis of COPD (AUC = 0.9207).

**Conclusion:**

Gut microbiota-targeted biomarkers represent potential non-invasive tools for the diagnosis of COPD.

## Background

Chronic obstructive pulmonary disease (COPD) is a progressive inflammatory disease affecting the airways, alveoli, and microvasculature. Its primary characteristics include irreversible airflow limitation, chronic lung inflammation, and remodeling of the small-airway compartment [Global Initiative for Chronic Obstructive Lung Disease (GOLD), 2021]. COPD is now a major public health problem all over the world and places a huge burden on economies, society, and medical care, especially in low- and middle-income countries [Halpin et al., 2019; Global Initiative for Chronic Obstructive Lung Disease (GOLD), 2021]. Our previous research showed the prevalence of COPD in people aged ≥40 years in China rose from 8.2% in 2007 to 13.7% in 2015, suggesting that 90 million Chinese adults might have this condition (Zhong et al., 2007; Wang et al., 2018). Owing to the absence of specific symptoms during the mild to moderate COPD (FEV1 ≥ 50% predicted, GOLD stages I-II) stages and because few people undergo a pulmonary function test before they are diagnosed with COPD (Wang et al., 2018), a large proportion of patients are often diagnosed when at an advanced stage of disease (GOLD stages III–IV) (Fang L. et al., 2018). Therefore, in addition to lung function testing, another non-invasive biomarker for the diagnosis of COPD is urgently needed.

The role of the gut microbiome in the development of this disease is now of considerable interest (Nie et al., 2019). Recent studies have linked changes in gut microbial composition and function with the development of COPD (Bowerman et al., 2020; Li et al., 2020, 2021; Lai et al., 2022). The gut microbiota and microbial metabolome of patients with COPD have been shown to be distinct to those of healthy individuals (Bowerman et al., 2020). In addition, the gut microbial composition has been shown to affect the development of cigarette smoke-induced COPD in mice, and the commensal bacterium *Parabacteroides goldsteinii* has been shown to ameliorate COPD (Lai et al., 2022). In our previous study, we showed that an abnormal gut microbiota in patients with COPD is associated with airway inflammation, and that the progression of COPD in mice is accelerated by fecal transplantation from mice with COPD, indicating a direct influence of the gut microbiota on COPD (Li et al., 2021). Furthermore, the concept of the use of the gut microbiome for the non-invasive diagnosis of colorectal cancer, hepatocellular carcinoma, and alcoholic liver disease has been validated in number of studies (Yu et al., 2017; Ren et al., 2019; Smirnova et al., 2020). However, the diagnostic potential of the gut microbiome for COPD has yet to be evaluated.

In the present study, we collected a total of 160 fecal samples from patients with COPD (GOLD stages I or II, *n* = 31; GOLD stages III or IV, *n* = 49) and healthy controls (*n* = 80) in Guangzhou city (Guangdong Province, P.R. China) and performed metagenomic sequencing of the microbial DNA content. The taxonomic and functional features of the microbiomes of patients with COPD and healthy controls were analyzed and used to evaluate the potential of the gut microbiome for use as a non-invasive biomarker of COPD.

## Methods

### Study design and participants

In the current study, participants were selected from our previous COPD cohort studies (Zhou et al., 2014, 2017; Liu et al., 2017; Li et al., 2019), which were funded by the National Key Research and Development Program of China (No. 2016YFC1304101). All patients were residents of Guangzhou and characterized by similar lifestyle and eating habits. Some of the most common foods in Guangzhou include rice, seafood, dim sum, cantonese roast meat and vegetables and herbs. The healthy control group was recruited from the same residential area. Following rigorous pathological diagnosis and exclusion procedures, a total of 160 fecal samples were obtained, with 31 from patients with COPD GOLD severity stages I or II, 49 from patients with COPD GOLD severity stages III or IV, and 80 from healthy controls. The Ethics Commission of the First Affiliated Hospital of Guangzhou Medical University approved the study (No. 2017–21, e-Appendix 1), and written informed consent was obtained from all participants. Participant clinical information, including sex, age, body mass index (BMI), smoking index, and spirometry data, was collected. The COPD Assessment Test (CAT) score and modified Medical Research Council (mMRC) Dyspnea Scale score were also calculated.

### Inclusion and exclusion criteria

In accordance with the GOLD guidelines [Global Initiative for Chronic Obstructive Lung Disease (GOLD), 2021], 80 male participants between 40–70 years of age were diagnosed with COPD. The participants included 39 individuals with COPD GOLD severity stages I or II (FEV1/FVC ratio < 70% and FEV1 50–80%), and 41 individuals with COPD severity stages III or IV (FEV1/FVC ratio < 70% and FEV1 < 50%). All the participants underwent chest X-ray, electrocardiography, abdominal ultrasonography, and blood, urine, and fecal tests. The exclusion criteria were: presence of other diseases, such as hypertension, cancer, diabetes and gastrointestinal disease; history of cystic fibrosis, asthma, and/or another clinically significant lung disease other than COPD; and treatment with a systemic corticosteroid and/or an antibiotic (inclusive of macrolide antibiotics) within the preceding 8 weeks. The exclusion criteria for the healthy controls were clinically significant lung disease, gastrointestinal disease, hypertension, diabetes, obesity, metabolic syndrome, and treatment with antibiotics within the preceding 8 weeks.

### Collection of fecal samples and DNA extraction

Fresh stool samples were collected from donors in the morning. The consistency of each sample was assessed using the Bristol Stool Form Scale, and only samples with types 2–5 were included. The stool samples were immediately placed in sterile containers and stored in −80°C freezers at the research laboratory until further processing. Bacterial DNA was extracted from 200 mg samples using a MagPure Stool DNA KF kit (Magen Biotechnology, Guangzhou, China), according to the manufacturer’s instructions. The DNA was quantified using a Qubit dsDNA BR Assay Kit (Thermo Scientific, Waltham, MA, United States) and a Qubit Fluorometer (Thermo Scientific, Waltham, MA, United States), following the manufacturer’s instructions.

### PCR amplification, library construction and metagenomic sequencing

The DNA quality was assessed through 1% agarose gel electrophoresis, and the DNA library was generated using the method previously outlined (Huang et al., 2017). In brief, genomic DNA (1 μg) was fragmented using covaris to obtain random fragments. Magnetic beads were used to select fragments with an average size of 300–700 bp. The selected fragments then underwent end-repair, 3′ adenylation, adapters-ligation, and PCR amplification. The PCR products were subsequently purified using Magnetic beads. To form the final library, the double-stranded PCR products were heat denatured and circularized using a splint oligo sequence. The resulting single-strand circular DNA (ssCir DNA) was qualified through quality control (QC) assessment. The qualified libraries were sequenced using the BGISEQ-500 platform (BGI, Shenzhen, China) (Huang et al., 2017; Fang C. et al., 2018). The Genome Sequence Archive at the BIG Data Center (https://bigd.big.ac.cn; Beijing Institute of Genomics (BIG), Chinese Academy of Sciences) has received the raw sequencing read data for all samples, which can be accessed under the accession number PRJCA013653.

### Sequencing data processing

The Sunbeam pipeline was used to process raw sequencing reads for the metagenome. During processing, Cutadapt (v.2.5) was used for quality filtering, Komplexity was used to filter out sequences of low complexity, and BWA (v.0.7.17) was used to filter out host reads by mapping to the human genome GRCh38 (Li and Durbin, 2009; Marcel, 2011; Clarke et al., 2019). MEGAHIT (v.1.1.3) with default parameters was used to assemble non-human reads (Li et al., 2015). Genes sequences were predicted from assembled contigs using Prodigal (Hyatt et al., 2012) and a non-redundant (nr) gene collection was created by de-replicate the gene sequences using CD-Hit (Li and Godzik, 2006) at 95% identity over 90% of the shorter ORF length (set as −c 0.95, −aS 0.9, −g 1, −d 0).

Gene-level taxonomic profiling was accomplished by aligning genes to a curated collection of 6,530 representative bacterial genomes sourced from NCBI Genbank (retrieved in May 2021) using BLASTn (−e 0.01). For each gene, the alignments with the top 10% highest scores were kept requiring a minimum identity of 65% and coverage of 80%. Gene taxonomy was determined by achieving a consensus of at least 50% above the similarity threshold for a specific rank, with phylum requiring ≥65%, genus requiring ≥85%, and species requiring ≥95% (Li et al., 2014; Dai et al., 2019). The last common taxonomy level shared by these high-scoring hits was assigned as the taxonomy of each gene. To perform functional annotation of genes, the KEGG database was aligned with using DIAMOND (v.0.9.32.133), and the best-hit with identity ≥30% and coverage ≥70% was selected (Buchfink et al., 2015; Dai et al., 2019). The number of mapped reads was subsequently adjusted to 3 million per sample to account for differences in sequencing depth. The abundance of each gene in each sample was normalized by mapping the reads using BBMap (v.38.44) and estimating the coverage using the jgi_summarize_bam_contig_depths script (Bushnell, 2014; Kang et al., 2015). The gene-level abundances were then combined to obtain the KEGG orthologs (KOs).

### Statistical analysis

Alpha diversity was assessed using the Shannon index. Beta diversity was assessed using the Bray–Curtis dissimilarity index calculated from a weighted matrix abundance and visualized using principal coordinates analysis. The R software (R Project for Statistical Computing, Vienna, Austria) was used to perform the Wilcoxon rank-sum test to identify microbial taxonomic and functional features that exhibited differential abundance among the groups of individuals. Random forest analysis was performed using the differentially represented microbiome features in an attempt to predict the presence of COPD GOLD I or II grades and COPD GOLD III or IV grades, using five-fold cross-validation, and with the number of trees and mtry parameters (a tuning parameter in the random forest algorithm) defined using a grid search algorithm in the R caret package (Kuhn, 2008). Feature selection was performed according to the scheme in Feng et al. (2015). Specifically, the microbial taxonomic and functional features were ranked by their variable importance and sequentially added into the model. The cross-validation errors were averaged and plotted against the number of genes. The cutoff for feature selection was determined as the minimum error in the averaged curve plus the standard deviation at that point. Features with an error less than the cutoff were listed, and the optimal set was chosen as the set with the smallest number of features. *p*-values were adjusted for multiple hypothesis testing using the Benjamini-Hochberg procedure.

## Results

### Characteristics of the participants

**Figure 1:**
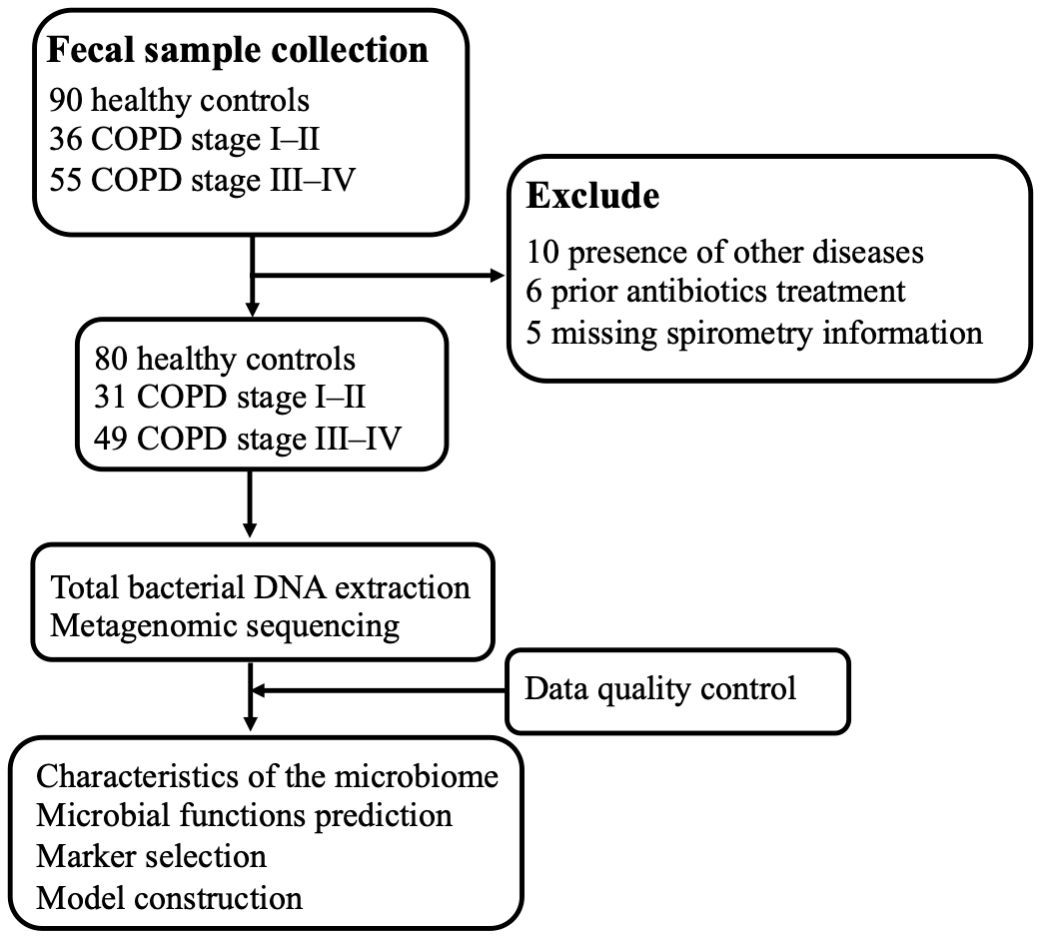
Schematic overview of the study workflow. A total of 181 fecal samples were obtained from 90 patients with COPD and 90 healthy controls. After a strict diagnosis and exclusion process, fecal samples remained from 31 participants from COPD stages I–II, 49 from participants with COPD stages III–IV, and 80 from healthy controls for DNA extraction, metagenomic sequencing, and data analysis. Finally, sequencing data from all the samples were used for bioinformatics analysis, and stratification analysis with respect to COPD was performed.

We collected 160 fecal samples (80 from controls and 80 from participants with COPD) from individuals resident in Guangzhou city. A total of 31 samples from participants with GOLD I or II COPD, 49 from those with GOLD III or IV COPD, and 80 from healthy controls were subjected to metagenomic sequencing after a strict diagnosis and exclusion process (Figure 1). Table 1 summarizes the characteristics of the participants. Participants in the COPD III–IV group had lower BMI and FEV_1_, FVC, and FEV_1_/FVC values, but higher COPD assessment scores, than those in the other groups.

**Table 1:** Demographic characteristics of the participants.

Characteristic	Healthy (*n* = 80)	COPD I-II (*n* = 31)	COPD III-IV (*n* = 49)	*p* value
Age, years	62.48 ± 5.75	64.13 ± 3.14	62.20 ± 6.00	NS
BMI, kg/m^2^	23.32 ± 3.13	21.37 ± 2.48	20.36 ± 2.72	<0.01^a^^b^
Smoking index (pack-yr)	38.81 ± 28.38	46.75 ± 29.28	42.02 ± 26.90	<0.05^a^
Smoking status, *n* (%)				<0.01^a^^b^
Never smoker	60 (75.0%)	0	0	
Current smoker	15 (18.8%)	24 (77.4%)	30 (61.2%)	
Ex-smoker	5 (6.2%)	7 (22.6%)	15 (38.8%)	
MMRC score	–	1.0 (0.0,1.0)	3.0 (2.0,3.0)	<0.001^c^
FEV_1_ (L)	2.54 ± 0.42	2.09 ± 0.46	0.96 ± 0.28	<0.001^a^^b^^c^
FEV_1_%	99.57 ± 14.2	81.10 ± 16.38	35.15 ± 8.96	<0.001^a^^b^^c^
FVC (L)	3.30 ± 0.54	3.46 ± 0.51	2.33 ± 0.52	<0.001^b^^c^
FVC%	102.94 ± 15.60	105.08 ± 13.10	68.68 ± 14.60	<0.001^b^^c^
FEV_1_/FVC%	76.99 ± 4.62	60.13 ± 8.17	42.12 ± 9.41	<0.001^a^^b^^c^

Data are shown as mean ± SD or median (interquartile range). Differences among continuous datasets were evaluated using ANOVA, with the Bonferroni correction. FEV_1_, forced expiratory volume in 1 s; FVC, forced vital capacity; BMI, body mass index; NS, no significant difference; COPD, chronic obstructive pulmonary disease; CAT, COPD assessment test; MMRC, modified Medical Research Council.

a *p* < 0.05 for the COPD I–II group vs. healthy controls.

b *p* < 0.05 for the COPD III–IV group vs. healthy controls.

c *p* < 0.05 for the COPD III–IV group vs. the COPD I–II group.

### Gut microbial composition of patients with COPD and healthy controls

**Figure 2:**
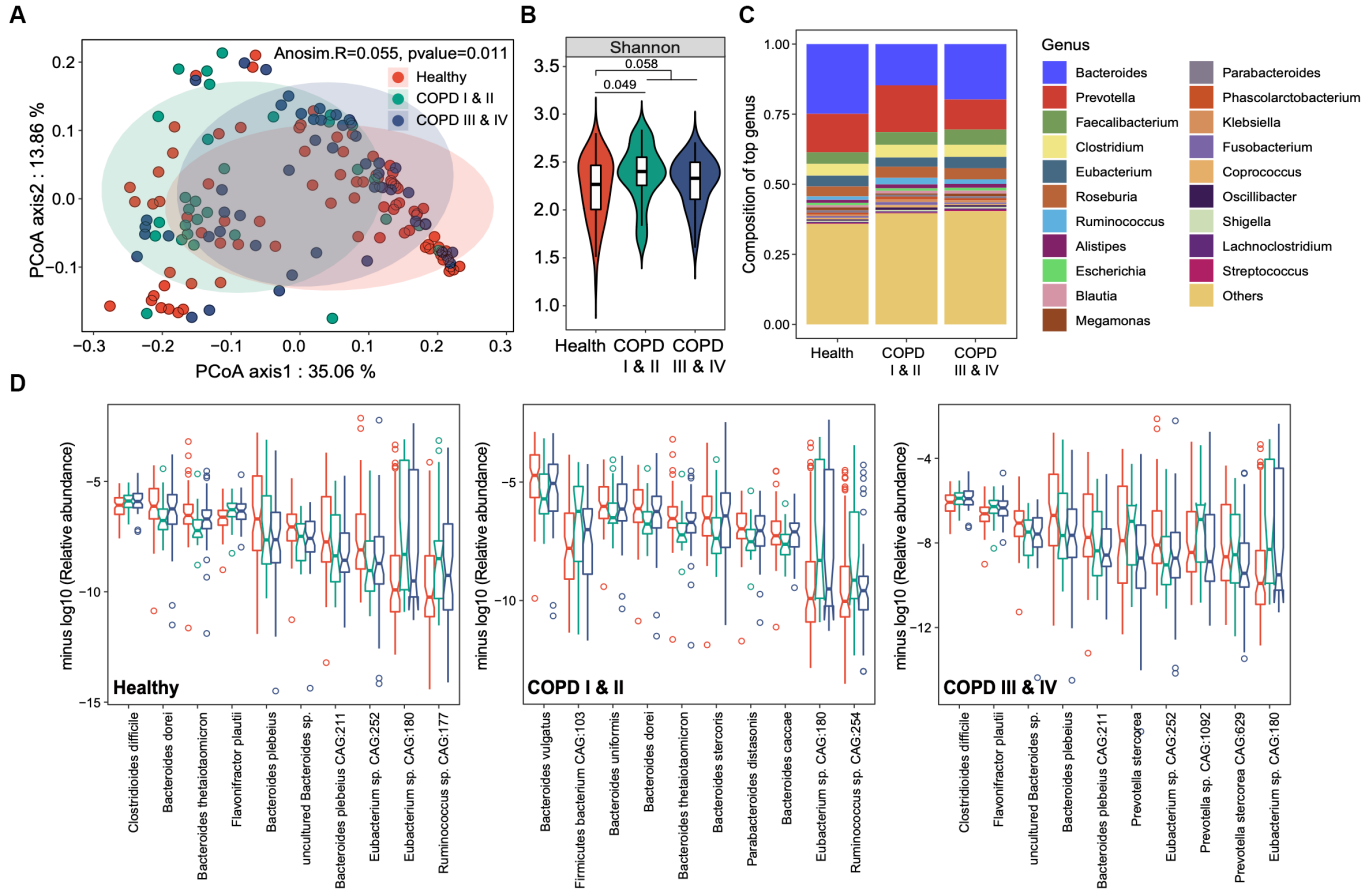
Gut microbial taxonomic profiles of healthy individuals and participants with COPD I or II and III or IV. **(A)** Principal coordinates analysis for the gut microbiome taxonomic profiles of the three groups. **(B)** Boxplots showing the alpha diversity (Shannon index) of the three groups. **(C)** The genus-level taxonomic profiles of the gut microbiomes of the three groups. The top 20 most abundant genera are shown. **(D)** Notch plots showing the relative abundances of the top 10 differentially abundant species-level taxa for the healthy controls, and participants with COPD I or II and III or IV. The minus log10 relative abundance is shown for each species.

We found a small but significant difference in the beta diversity of the gut microbiota among the groups (Adonis R = 0.055, *p* = 0.011, Figure 2A). The microbiome of the healthy controls had lower alpha diversity than either group of participants with COPD, and the alpha diversity was highest in the participants with COPD I or II (Figure 2B). *Bacteroides* was the most abundant genus in the cohort overall (21.3%), followed by *Prevotella* (13.4%), *Faecalibacterium* (4.6%), and *Clostridium* (4.3%) (Figure 2C). Differential analysis identified 21, 24, and 11 microbial species that were significantly enriched or depleted in the healthy, COPD I or II, and III or IV groups, respectively (FDR < 0.1, Figure 2D). Specifically, *Clostridioides difficile*, a well-established pathogen in the gut, was most abundant in participants with GOLD III or IV (0.324%) and least abundant in healthy individuals (0.242%, *p* = 0.004). The same trend was also identified for *Flavonifractor plautii* (0.15% in healthy and 0.2% in COPD, *p* = 6.08 × 10^−4^) and *Ruminococcus* sp. *CAG:177* (0.09% in healthy and 0.23% in COPD, *p* = 0.011). In contrast, *Bacteroides plebeius* and an unclassified *Bacteroides* sp. were most enriched in the healthy group and most depleted in the participants with GOLD III or IV (*p* < 0.001). Other members of the *Bacteroides* genus (*Bacteroides dorei*, *Bacteroides thetaiotamicron*, and *Bacteroides caccae*) were most depleted in the participants with GOLD I or II, whereas *Prevotella stercorea* and an unclassified *Prevotella* sp. were most enriched in this group (*p* < 0.05).

### Gut microbiome functions in participants with COPD and healthy controls

**Figure 3:**
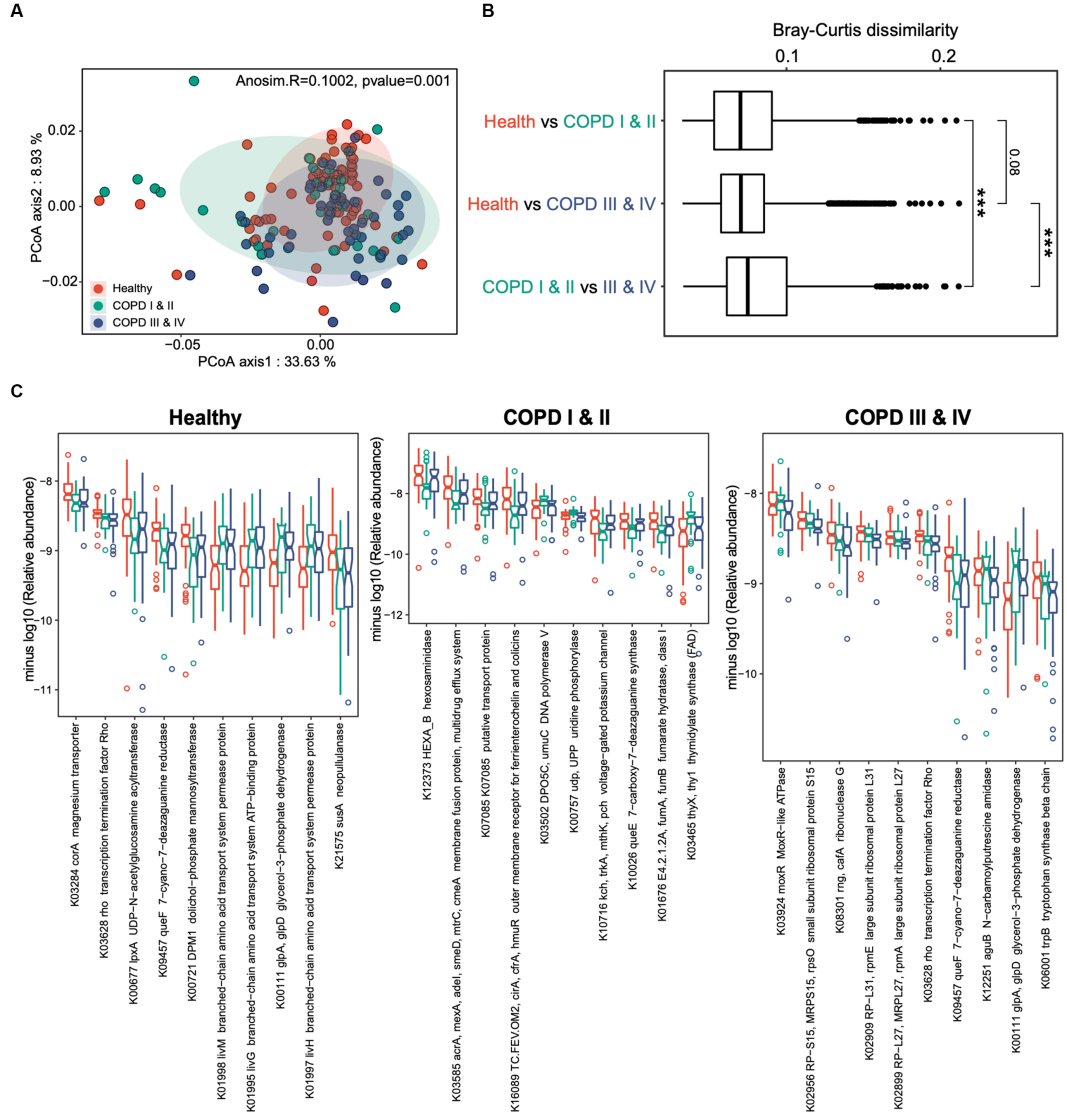
Gut microbiome functional profiles of healthy individuals, and participants with COPD I or II and III or IV. **(A)** Results of principal coordinates analysis for the gut microbiome taxonomic profiles of the three groups. **(B)** Boxplots showing the Bray–Curtis dissimilarity indices for the pairwise comparison of the healthy control versus COPD I or II groups, healthy control versus COPD III or IV groups, and COPD I or II versus III or IV groups. **(C)** Notch plots showing the relative abundances of the top 10 differentially abundant KEGG orthologs (KOs) for the healthy control, COPD I or II, and III or IV groups. The minus log10 relative abundance is shown for each KO.

We next assessed the differences in microbial functions among three groups. Functional profiling yielded 7,261 KEGG orthologs (KOs) in the cohort as a whole, spanning 396 functional categories. Principal components analysis revealed greater differences in gut microbial functions among the three groups compared to those identified based on taxonomic composition (Adonis R = 0.1, *p* = 0.001, Figure 3A). As for the gut microbiome composition, the gut microbiome functions of the participants with COPD I or II differed from those of the other two groups (Figure 3A). This was corroborated by the finding that the largest Bray–Curtis dissimilarity index was for the pairwise comparison between the COPD I or II and III or IV groups (*p* < 0.001, Figure 3B), which suggests the presence of substantial gut microbial dysbiosis in mild to moderate COPD.

Comparisons of the microbial functional KOs among the three groups identified 387, 188, and 131 genes that significantly differed in abundance in the healthy, COPD I or II, and III or IV groups (FDR < 0.05, Figure 3C). Interestingly, a large proportion of the KOs that substantially differed in abundance among the three groups were in participants with GOLD I or II, implying that gut dysbiosis is a particular feature of mild to moderate COPD. A large proportion of these were bacterial transporter genes, which were depleted (K03284) or enriched (K01998, K01995, and K01997) in participants with GOLD I or II, and this may play a role in the pathogenesis of the disease. Collectively, these results suggest that there are both compositional and functional alterations in the gut microbiomes of patients with COPD and that these are most pronounced in patients with COPD GOLD grades I or II.

### Gut microbial markers that differentiate patients with COPD from healthy controls

**Figure 4:**
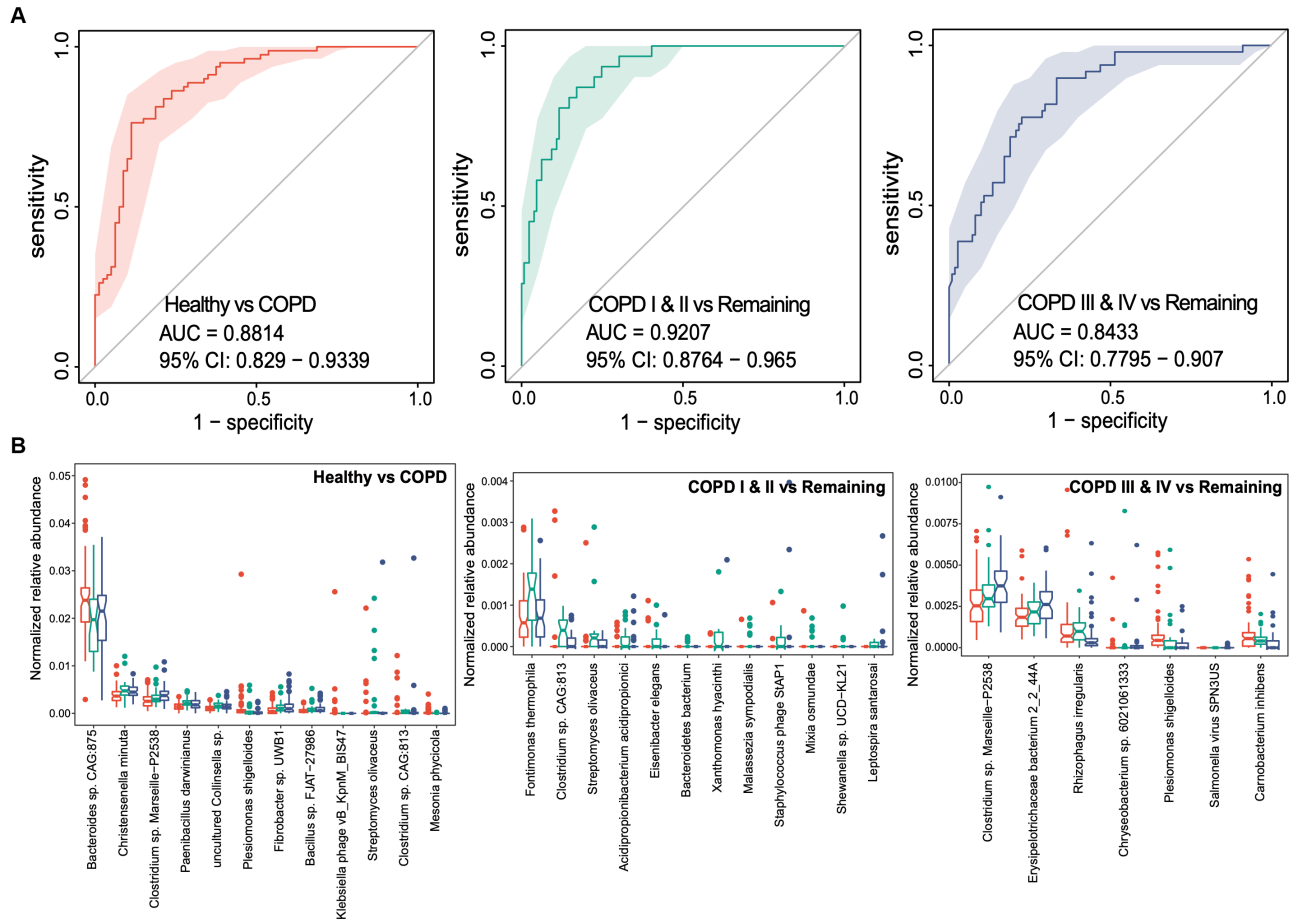
Random forest classifiers for the prediction of healthy status, COPD I or II, and III or IV using gut microbiome taxonomic features. **(A)** Receiver operating characteristic curve (ROC) for the classifiers for healthy status, COPD I or II, and COPD III or IV. The 95% confidence interval is shown for each ROC. **(B)** Notch plots showing the relative abundances of the microbial species retained in each classifier. The arcsin square root-normalized relative abundance is shown for each species.

Having established that the participants with COPD had gut microbial dysbiosis, we next aimed to determine whether the gut microbiota could provide non-invasive markers of particular stages of COPD. We performed random forest analyses to identify the gut microbial taxonomic and functional markers that were predictive of membership of each of the three groups. Using species-level taxonomic profiles, the areas under the receiver operating characteristic curves (AUCs) were found to be 0.881 (95% CIs: 0.829–0.934), 0.921 (95% CIs: 0.876–0.965), and 0.843 (95% CIs: 0.780–0.907) for the classification of healthy individuals, participants with COPD I or II, and those with COPD III or IV, respectively, indicating high levels of performance for the prediction of healthy status and the two COPD groups (Figure 4A). After feature selection based on variable importance, 12, 12, and 7 species-level taxa were retained in the classifier for each of the three groups (Figure 4B). To distinguish patients with COPD from healthy controls, *Bacteroides* sp. CAG875, *Christensenella minuta*, and *Clostridium* sp. Marseille-P2538 were the three most abundant species retained in the classifier. *Fontimonas thermophile*, *Clostridium* sp. CAG813, and *Streptomyces olivaceus* were the three most abundant species in the classifier for participants with COPD I or II, and *Clostridium* spp. Marseille-P2438, *Erysipelotrichaceae* bacterium, and *Rhizophagus irregularis* were the most abundant species that distinguished participants with COPD III or IV from the others.

**Figure 5:**
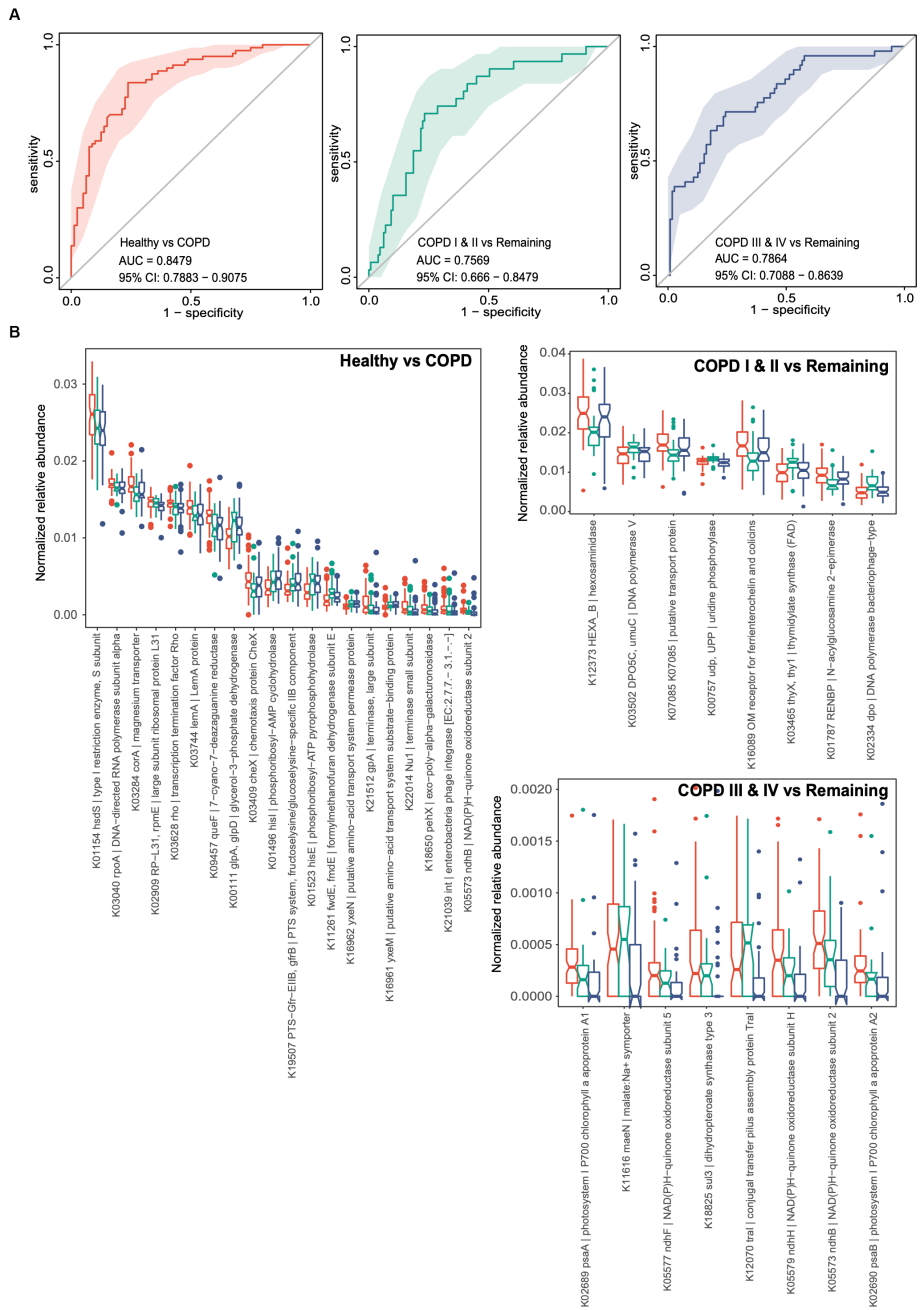
Random forest classifiers for the prediction of healthy status, COPD I or II, and III or IV using gut microbiome functional genes. **(A)** Receiver operating characteristic curves (ROCs) for classifiers for the prediction of healthy status, COPD I or II, and COPD III or IV. The 95% confidence interval is shown for each ROC. **(B)** Notch plots showing the relative abundances of the KOs retained in each classifier. The arcsin square root-normalized relative abundance is shown for each KO. The top 20 differential KOs are shown for the classifier for healthy status versus COPD.

We performed similar random forest analyses using gut microbial functional KOs. The model generated performed less well than the one discussed above, with AUCs of 0.848, 0.757, and 0.786, respectively, for each of the three groups (Figure 5A). Thirty-two KOs were retained in the classifier for the predictor of healthy individuals after feature selection, while small sets of 8 and 8 KOs were retained in the classifiers for participants with GOLD I or II and GOLD III or IV (Figure 5B). Consistent with the feature selection results, all these KOs were differentially abundant between the corresponding groups of comparison. Taken together, these results suggest the potential for the use of gut microbial taxonomic and functional components as non-invasive biomarkers for the identification of patients with mild to moderate COPD or later-stage COPD.

## Discussion

The potential involvement of the gut microbiome in the pathogenesis of COPD has gained considerable attention in recent years (Bowerman et al., 2020; Kotlyarov, 2022). In our prior investigation, we detected microbial dysbiosis in the gut microbiota of COPD patients and a plausible association between gut microbial dysbiosis and the advancement of COPD (Li et al., 2021). In the present study, we developed a diagnostic model comprising gut microbial features and clinical variables. The results suggest that there are significant differences in both gut microbial compositional and function between patients with COPD and healthy individuals. Gut microbiota-related biomarkers may therefore represent potential non-invasive tools for the diagnosis of mild to moderate COPD and later-stage COPD.

The impact of the gut microbiota extends beyond the intestinal tract and has been implicated in the progression of various lung diseases, such as lung cancer, asthma, and pneumonia (Depner et al., 2020; Stevens et al., 2022; Zhao et al., 2022). Several recent studies have established a direct association between the gut microbiota and COPD (Bowerman et al., 2020; Kotlyarov, 2022; Lai et al., 2022). The alterations to the gut microbial composition associated with poor lung function in patients with COPD were first reported by Bowerman et al., who observed a lower abundance of the family Lachnospiraceae and a higher abundance of *Streptococcus vestibularis* (Bowerman et al., 2020). In 2021, Hsin-Chih et al. reported that the composition of the gut microbiota has a significant impact on the development of COPD induced by cigarette smoke, and that fecal microbiota transplantation can restore the pathogenesis of COPD. We previously found that the gut microbiota of patients with COPD is characterized by lower microbial abundance and diversity, a distinct overall composition of the microbiota, a *Prevotella*-dominated gut enterotype, and low concentrations of short-chain fatty acids (Li et al., 2021). Moreover, gut microbial dysbiosis is associated with the progression of COPD in an animal model (Li et al., 2020, 2021). In the present study, we present compelling evidence that the gut microbiome composition and function is altered in COPD. We found that *Clostridioides difficile*, *Flavonifractor plautii*, and *Ruminococcus* spp. were the most abundant species in fecal samples from patients with COPD III or IV, and were least abundant in healthy individuals. In addition, *Bacteroides plebeius* and an uncharacterized *Bacteroides* sp. were least abundant in patients with COPD III or IV.

However, the functions of these microbial species in patients with COPD are currently unclear. *Flavonifractor plautii*, a gram-positive anaerobic bacterium that belongs to the Clostridium genus, has been found in human feces and has been demonstrated to metabolize catechins. Several studies have shown that *F. plautii* is capable of inhibiting inflammation. For instance, Mikami et al. reported that the oral administration of *F. plautii* strongly suppresses Th2 immune responses in mice (Mikami et al., 2021). However, recent studies have shown that *F. plautii* is enriched in early-onset colorectal cancer (Kong et al., 2023). As for *Clostridioides difficile*, a well-established pathogenic gut bacterium, is a major cause of hospital-acquired infection, which cause intestinal epithelial injury and inflammation and even drive colonic tumorigenesis (Guh et al., 2020; Drewes et al., 2022). When *Bacteroides plebeius*, a seaweed-degrading species, is depleted, glycosaminoglycan metabolism is downregulated, which predisposes to damage to articular cartilage in rheumatoid arthritis (Cheng et al., 2022). Previous studies have also shown that the abundance of *B. plebeius* is affected by the diet; for example, *B. plebeius* s-OTU is positively associated with brown or wild rice consumption and negatively associated with the consumption of some processed meat (Peters et al., 2020). *Ruminococcus* spp., which degrade dietary nutrients, providing the host with energy and nutrients, are also considered to be proinflammatory microbes. For example, Szu-Ju et al. reported that the abundance of *Ruminococcus spp. is* significantly associated with low fecal concentrations and high plasma concentrations of SCFAs in patients with Parkinson’s disease (Chen et al., 2022). In the present study, we observed the enrichment of potential pathogens, such as *Flavonifractor plautii*, *Bacteroides stercoris*, *Clostridioides difficile*, and pro-inflammatory *Prevotella spp*. Additionally, we noted a depletion of commensal bacteria, including *Bacteroides vulgatus*, *Eubacterium spp.,* and *Bacteroides plebeius*.

A number of compelling studies have established that metagenomic analysis of the gut microbiome identifies potentially useful non-invasive biomarkers for diseases of the digestive system and others (Yu et al., 2017; Zheng et al., 2020; Coker et al., 2022). In the present study, we found that the microbial genera associated with COPD are an excellent means of differentiating patients with COPD from healthy individuals, with an AUC of 0.8814 (95% confidence interval (CI): 0.8290–0.9339). In addition, the AUC was 0.9207 (95% CI: 0.8764–0.9650) for the differentiation of mild to moderate COPD (COPD I or II), which implies that gut microbial markers would provide an accurate method for the early diagnosis of COPD. Thus, specific alterations in gut microbiota might have a future as non-invasive biomarkers of COPD.

We acknowledge the following limitations of the present study. Firstly, the sample size was relatively small, and we only recruited Chinese participants. Therefore, further large-scale, multi-center studies involving participants of diverse ethnicities should be conducted to validate the generated model. Furthermore, it is necessary to establish a link between the differences observed in each stage and the potential significance of diagnosing this disease through metagenomic profiling. Secondly, the present study was cross-sectional in nature; therefore, we cannot infer causal relationships between the gut microbiota and COPD, nor can we comment on the mechanisms involved or the specific effects of differential microbial species abundance.

## Conclusion

Taken together, the present findings suggest the gut microbial taxonomic and functional components may represent useful non-invasive biomarkers for the differentiation of healthy individuals, and those with mild to moderate COPD or later-stage COPD.

## Data availability statement

The datasets presented in this study can be found in online repositories. The names of the repository/repositories and accession number(s) can be found below: https://bigd.big.ac.cn, PRJCA013653.

## Ethics statement

The studies involving human participants were reviewed and approved by ChiCTR-IIR-17012604. The patients/participants provided their written informed consent to participate in this study.

## Author contributions

NL, PR, YZ, and BL were responsible for the study concept, project direction, and experiment design. NL, CC, ZLD, ZSD, and JP acquired the samples and clinical information, analyzed the results, and drafted the manuscript. ZW and XY conducted the microbial sequencing analysis. All authors contributed to the article and approved the submitted version.

## Funding

Funding for this work was provided by the National Natural Science Foundation of China (grant numbers 81970045 and 81900030), the Local Innovative and Research Teams Project of Guangdong Pearl River Talents Program (grant number 2017BT01S155), the Guangdong Province Key Field R&D Program (grant number 2020B1111330001), the Foundation of the National Key Laboratory of Respiratory Diseases (grant number SKLRD-Z-202308), and the Science and Technology Program of Guangzhou (grant number 202201020434).

## Conflict of interest

The authors declare that the research was conducted in the absence of any commercial or financial relationships that could be construed as a potential conflict of interest.

## Publisher’s note

All claims expressed in this article are solely those of the authors and do not necessarily represent those of their affiliated organizations, or those of the publisher, the editors and the reviewers. Any product that may be evaluated in this article, or claim that may be made by its manufacturer, is not guaranteed or endorsed by the publisher.

## Abbreviations

AUC: Area under the curve
COPD: Chronic obstructive pulmonary disease
CAT: COPD assessment test
FEV_1_: Forced expiratory volume in 1 s
FVC: Forced vital capacity
FEV_1_/FVC: Forced expiratory volume in 1 s to forced vital capacity ratio
MMRC: Modified Medical Research Council
OTU: Operational taxonomic unit
